# Supplementary material for: Ultra-compact broadband terahertz spectroscopy sensor enabled by resonant-gradient metasurface
Source: Nat Commun. 2025 Dec 11;16:11462. doi: 10.1038/s41467-025-66310-w (PMC12748858; doi:10.1038/s41467-025-66310-w)
Supplement: Supplementary file 1 — Supplementary Information [file 41467_2025_66310_MOESM1_ESM.pdf]

Ultra-compact broadband terahertz spectroscopy sensor enabled by resonant-gradient metasurface

Ride Wang[1,*,#], Dongze Zhang[2,#], Lu Chen[3,#], Nan Zhang[4,#], Dongxiao Li[5], Rundong Jiang[1], Xiaobao Zhang[1], Xiao Yang[1], Liuyang Zhang[4], Shuming Wang[6,*], Xiaogang Liu[7,*], Chao Chang[1,2,*], Din Ping Tsai[8,*]

[1] *Innovation Laboratory of Terahertz Biophysics National Innovation Institute of Defense Technology Beijing 100071, P. R. China*

[2] *School of Physics, Peking University Beijing 100871, P. R. China*

[3] *Key Laboratory of Weak-Light Nonlinear Photonics, Ministry of Education, School of Physics, Nankai University, Tianjin 300071, China*

[4] *School of Mechanical Engineering, Xi'an Jiaotong University, Xi'an, Shaanxi 710049, PR China.*

[5] *Key Laboratory of Optoelectronic Technology & Systems of Ministry of Education, International R & D center of Micro-nano Systems and New Materials Technology, Chongqing University, Chongqing 400044, China*

[6] *National Laboratory of Solid-State Microstructures, School of Physics, Nanjing University, Nanjing, 210093, China*

[7] *Department of Chemistry, National University of Singapore, Singapore, Singapore.*

[8] *Department of Electrical Engineering and State Key Laboratory of Optical Quantum Materials, City University of Hong Kong, Kowloon, Hong Kong SAR 999077, China.*

[*] *Corresponding authors: dptsai@cityu.edu.hk, gwyzlzssb@pku.edu.cn, chmlx@nus.edu.sg, wangshuming@nju.edu.cn, wangride@mail.nankai.edu.cn*

[#] *These authors have equal contribution for the manuscript.*

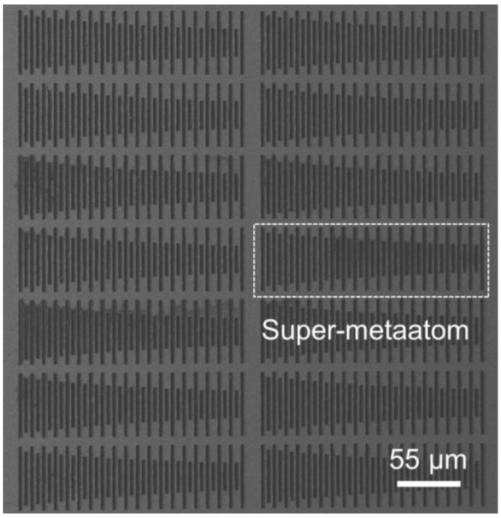

Figure S1. SEM images of super-metaatoms composed of THz plasmonic gradient gold microbars. The design parameters are detailed in Table S1. Scale bar: 55 μm.

Table S1. Design parameters of microbars within a super-metaatom

Microbar lengths (μm)	110 93 110 89 110 85 110 81 110 77 110 74 110 71 110 68 110 65 110 63 110 61 110 59 110 57 110 55 110 53 110 51 110 50 110 49 110 48 110

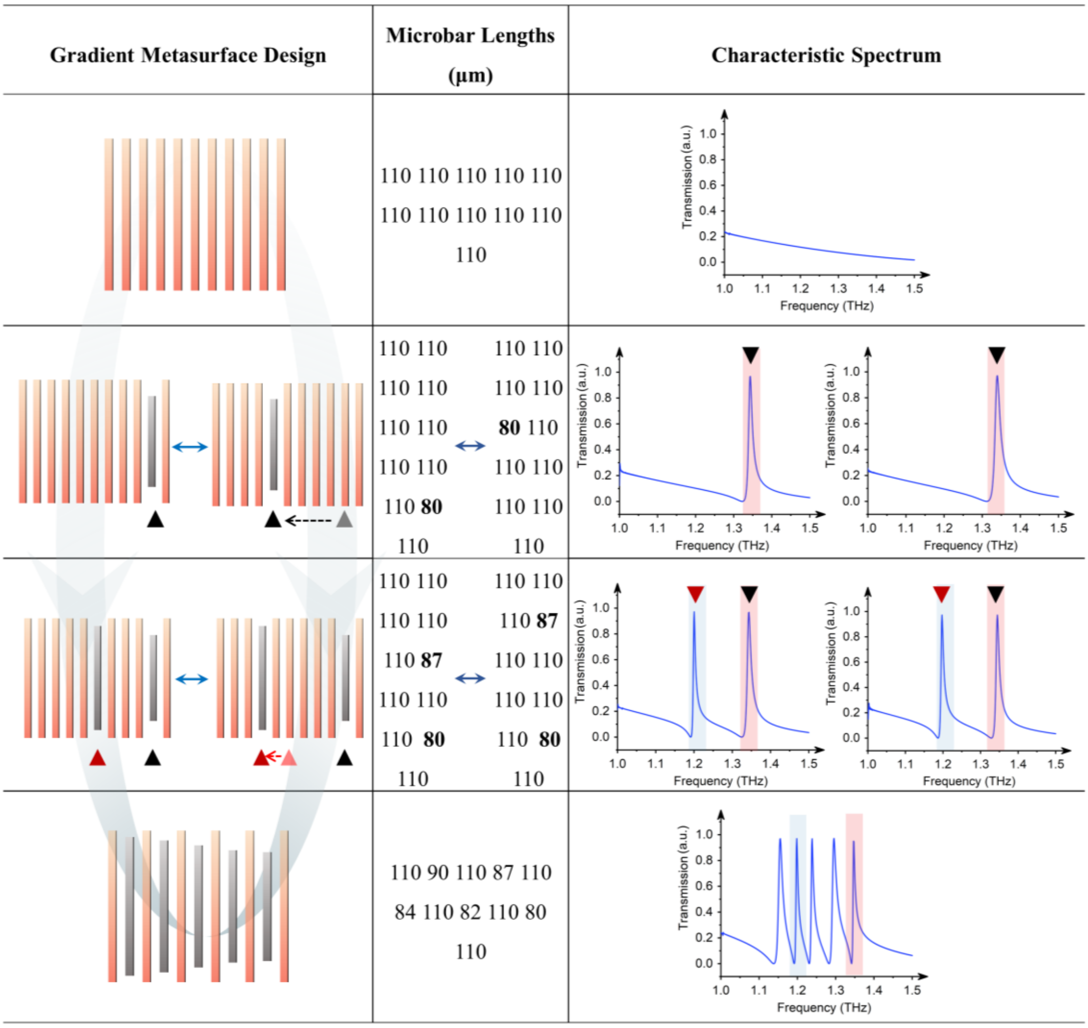

Figure S2 Design process for gradient metasurface supporting broadband enhanced THz spectroscopy. Our design consists of a series of gold microbars. When all microbars are of the same dimensions (as shown in the first panel of Figure S2), each microbar acts as a bright dipole mode and there is no coupling between neighbors. Then, we introduce defects at arbitrary positions in the microbar array and a QBIC state is excited with a finite and high Q-factor. As the number of defects introduced in the structure increases, the QBICs states are excited at different resonant frequencies, eventually offering on-demand coverage of the broadband spectrum. The difference between the defects (varying the length of the microrods) determines the spacing between resonant frequencies.

Table S2. The list for the performance of various THz metasensors including the Concept of the Work, Operating Frequency, Single/Multiple/Continuous Band(s), Metal/Dielectric, Integrated or not, Real Time or not, Analyte State, Mixed/Single Analyte(s), Quantity/Quality (Quant./Qua.), and Relative Spectral Efficiency.

No	Literature	Concept of the Work	Detected Mater. (Operating Frequency)	Single/ Multiple/Continuous Band(s)	Metal/ Dielectric	Integrated	Real Time	Analyte State (liquid (L.) /Solid (S.))	Mixed /Single Analyte(s)	Quantity/Quality	Relative Spectral Efficiency ($\Delta f/(f_{centra}\cdot D)$)
**1**	*Science* ***2018,*** *360, 1105–1109.*	Pixelated dielectric metasurfaces	Protein A/G, a mixture of PMMA and PE polymers, and glyphosate pesticide **(40.47-52.46 THz)**	Multiple	**Dielectric**	×	×	S.	Mixed	Quant. & Qua.	**0.258 mm^{-2}**
**2**	*Nat. Photon. 13, 390-396(20* ***19****).*	Pairs of tilted silicon nanobars	M-IgG, R-IgG **(360 THz)**	Single	**Dielectric**	×	√	L.	Mixed	Quant.	/
**3**	*Science Adv. 5, eaaw2871(201* ***9****)*	A zigzag array of elliptical germanium resonators On a calcium fluoride substrate	Proteins, aptamers, polylysine **(33 – 54 THz)**	Multiple	**Dielectric**	×	×	S.	Mixed	Qua.	/
**4**	*Nat. Commun. 2018, 9 (1), 2160.*	Plasmonic self-similar overlapping nanoantenna arrays	Biomimetic lipid membranes with different polypeptides as well as the dynamics of vesicular cargo release **(30-120 THz)**	Double	**Metal**	√	√	L.	Mixed	Quant. & Qua.	/
**5**	*Nat. Commun. 13, 3470(2* ***022****).*	Splitting Resonators	Pathogenic bacteria **(0.8 THz)**	Single	**Metal**	√	×	S.	Mixed	Qua.	/

**6**	*Nat. Nanotech. 19, 1804–1812, (2024).*	Dielectric dual-**gradient metasurfaces**	PMMA **(41.67-53.57 THz)**	**Continuous** broadband	**Dielectric**	×	×	S.	Single	Qua.	**0.926 mm^{-2}**
**7**	*Adv. Mater.* ***2024****, 2314279 (2024).*	Dielectric **gradient metasurfaces**	PMMA, biological analyte **(29.98-95.93 THz)**	**Continuous** broadband	**Dielectric**	×	×	**S. & L.**	Single	Quant. & Qua.	**0.63 mm^{-2}**
**8**	*ACS Nano* ***2024*** *18 (18), 11644-11654*	Pixelated dielectric metasurface	Photoswitchable AzoPC lipid membranes **(41.97-53.96 THz)**	Multiple	**Dielectric**	**√**	**√**	**L.**	Single	Quant. & Qua.	**0.125 mm^{-2}**
**9**	*Adv. Mater.* ***2023****, 2307494*	Resonance-gradient metasurfaces	\ **(71.43–120 THz)**	**Continuous** broadband	**Dielectric**	×	×	\	\	**Qua.**	**2.623 mm^{-2}**
**10**	*Adv. Mater.* ***2025****, 2418147*	**Multi-QBIC resonant metasurface**	Neurotransmitter molecules (L-glutamate, γ-aminobutyric acid/GABA) **(1.1–2.1 THz)**	Multiple	**Metal**	**√**	**√**	**S. & L.**	Mixed	**Quant. & Qua.**	**5.68 mm^{-2}**
**11**	***Our Work***	**Plasmonic resonant gradient metasurfaces**	**Neurotransmitters and their metabolic intermediates (1.2 – 2.1 THz)**	**Continuous broadband**	**Metal**	**√**	**√**	**S. & L.**	Mixed	**Quant. & Qua.**	**10.91 mm^{-2}**

D is the area of the metasurface region.

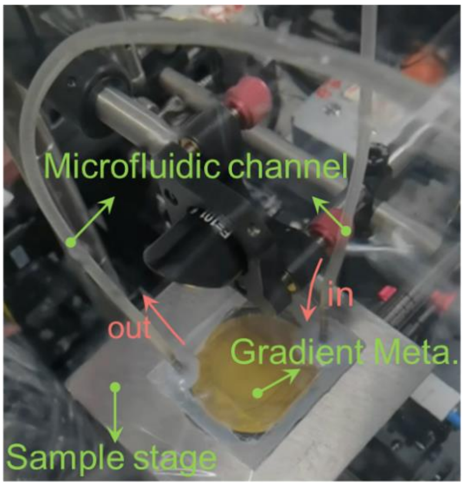

Figure S3 The experimental setup for real-time dynamic detection of metasensors utilizing wideband THz spectroscopy achieved through the combination of microfluidic channels and resonant gradient metasurfaces.

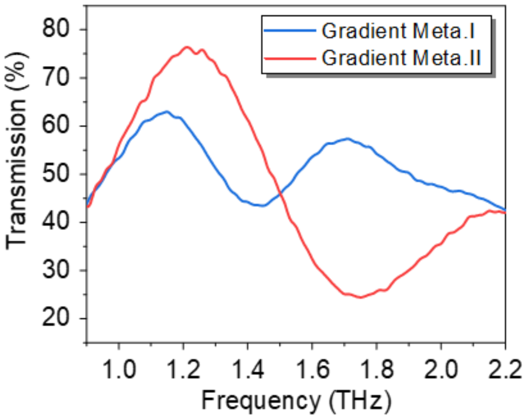

Figure S4 Experimental transmission spectra of two representative super-metaatoms with different design parameters. By judiciously varying the tuning size and the variation rate, application-specific coverage of the transmission spectra can be tailored. The design parameters are detailed in Table S3.

Table S3. Design parameters of microbars within a super-metaatom

Super-metaatom	Microbar lengths (μm)
I	110 90 110 87 110 84 110 82 110 80 110 78 110 76 110 74 110 72 110
II	110 70 110 68 110 66 110 64 110 62 110 60 110 58 110 56 110 54 110 52 110

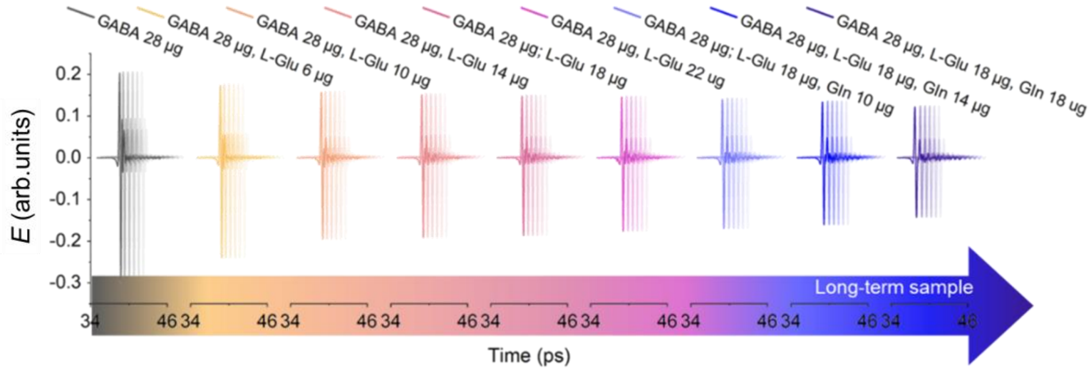

Figure S5 Similar to Figure 4d, the schematic shows the stabilized signal after continuous acquisition of the time-domain signal under different scenarios. Figure 4i was obtained by performing a Fourier transform on the signal window (34 - 46 ps) of the time-domain signal.

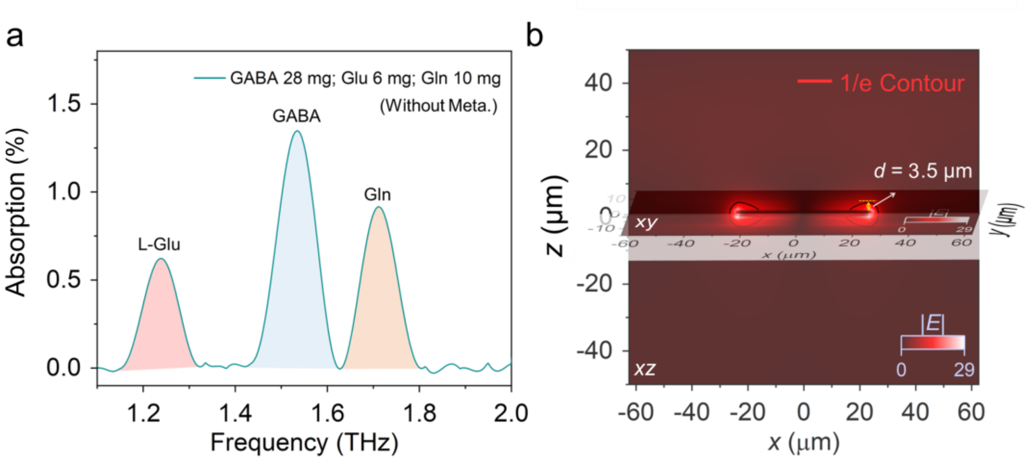

Figure S6 (a) Absorption spectrum calculated from the transmission spectra in Figure 4h. (b) The electric field distribution and intensity in both the *xy* and *xz* planes of the structural unit (L_1 = 48 μm) were monitored separately. The black solid line represents the 1/e contour of the maximum enhanced electric field. d = 3.5 μm is the height from the position of the 1/e electric field intensity to the metasurface surface.

To compute the enhancement factor (EF), we first extract the absorption intensities at the target frequency (1.54 THz for GABA). For instance, if the reference absorption I_{ref} is 1.35% (Figure S6a) and the sensor-enhanced absorption I_{ETHzAS} is 1.72% (Figure 4k), the signal enhancement $I_{\text{ETHzAS}} - I_{\text{ref}}$ equals 0.37%. To calculate the molecular ratio, we compare the effective interaction volumes under identical illumination conditions (same beam spot size), focusing exclusively on thickness differences. The reference volume V_{ref} spans the full microfluidic channel thickness (1 mm = 1000 μm), while the enhanced volume V_{ETHzAS} is now defined by the metasurface's interaction depth of d = 3.5 μm (Figure S6b). So, the EF is: $EF \approx 80$.

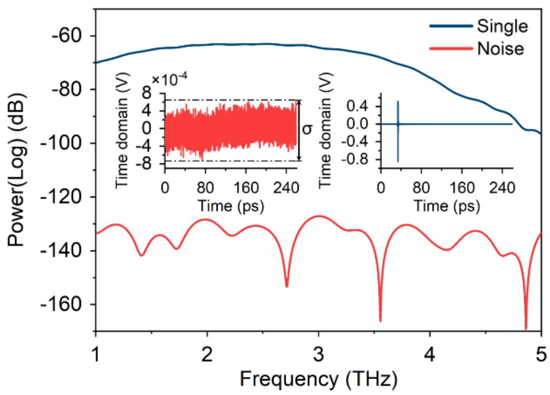

Figure S7 Power spectrum of the THz-TDS signal (blue) and noise floor (red), showing the dynamic range across the frequency range of 1 - 5 THz. (Inset on the right) Time-domain signal (blue line) with typical THz pulse oscillations, measured over a 260 ps delay range. The measurement was performed with 1028 averages to ensure data reliability. (Inset on the left) The noise in the time domain (red line), extracted from a flat region of the signal, highlighting the Noise Level (peak-to-peak) of 1.384 mV.

Time-domain signal and noise parameters (minimized by 3% humidity control).

Signal amplitude range: -0.8 V to +0.5 V (peak signal $E_{peak} \approx 0.8$ V).

Noise level (peak-to-peak): σ = 0.000653-(-0.000731) = 1.384 mV.

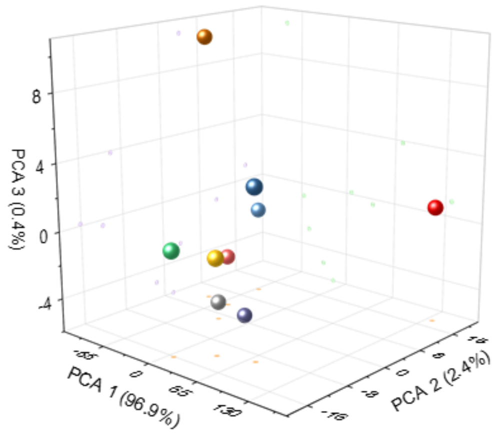

Figure S8 Principal Component Analysis (PCA) was applied to evaluate spectral weight scores in a reduced 2D space, verifying that the spectral data captured comprehensive biological characteristics. This analysis enabled sample clustering and visualization of correlations and distinctions, confirming the sufficiency of spectral data for representing inherent biological features. Due to the number of samples (n) being less than the number of features (421), the covariance matrix rank is constrained to n - 1. The first three principal components (PCs), accounting for the highest variance, were selected for visualization. Data were preprocessed, followed by covariance matrix computation, eigen decomposition, eigenvalue sorting, and projection onto the top-k eigenvectors. Note: PCA was used here solely for visualization to illustrate clustering patterns in 3D space. All subsequent modeling utilized the full 421-dimensional spectral data without dimensionality reduction.

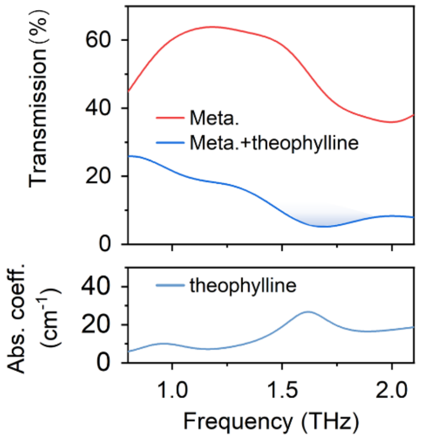

Figure S9 Absorption spectrum and identification results for the unknown sample (theophylline).

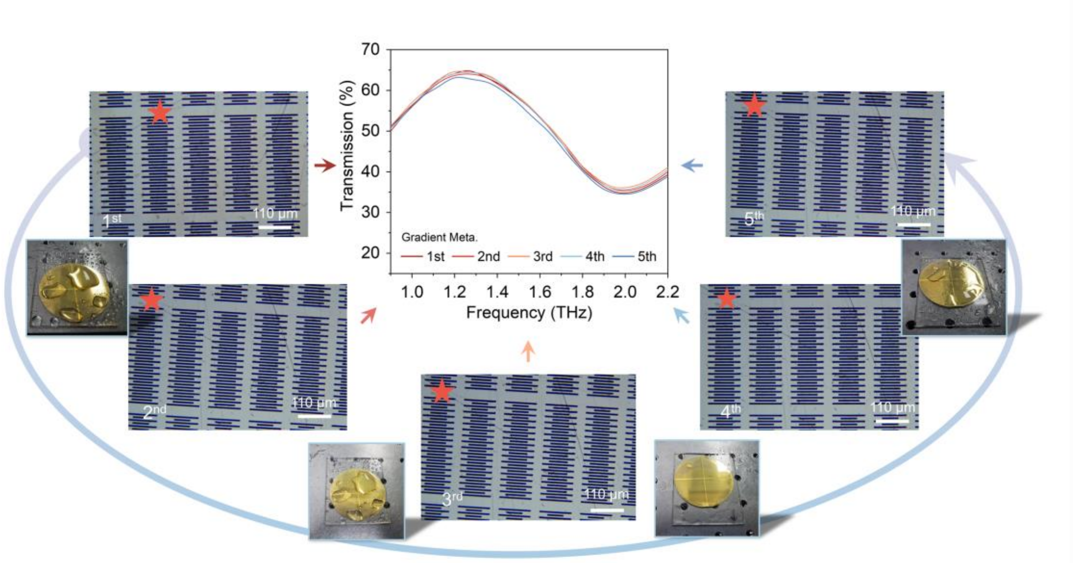

Figure S10 Stability testing results of the plasmonic gradient metasurface sensor under repeated measurements and solvent exposure. Schematic of the testing setup and comparison of transmission spectra after 5 cycles of microfluidic testing. Sample images show optical microscope images of the metasurface before and after cleaning for 5 cycles.
